# The Impact of Academic Publication: Inequity for Women in Behavior Analytic Journals

**DOI:** 10.3389/fsoc.2022.782914

**Published:** 2022-06-09

**Authors:** Jennifer M. Krebsbach

**Affiliations:** Education Department, California State University Sacramento, Sacramento, CA, United States

**Keywords:** authorship, editors, gender equity, higher education, sex, tenure, women

## Abstract

The number of women in higher education has increased over the past few decades but are still not at an equal level to their male counterparts, especially at the tenured level. One area of note within the tenure process is research. This area is highly valued by certain universities and could shed light on discrepancies in the number of female faculty as the faculty position becomes more prestigious. The author downloaded 21 years of publication data for seven prestigious behavior analytic journals and used quantitative methods to determine if the rates of publication differed between a previous study and today. There were 8,778 final articles yielding 27,225 authors in total. Data showed that women are represented more frequently overall, across time and all journals, less frequently in prestigious authorship positions, and more often when the sex of the editor at the time of publication was also female. While women's participation has increased over time, and since the original study, there is still disproportionate representation compared to the entirety of the field, in the order of authorship positions, and for editor-in-chief positions.

## Introduction

The first step to addressing inequity is to identify where it exists. In their study of women published in applied behavior analysis (ABA) journals, McSweeney et al. (2000) found that as few as 20% of published authors from 1978 to 1997 were female, indicating inequity between men and women publishing in the field. Although the numbers showed an increasing percentage of female authors across their research, the authors suggested two reasons as to why the discrepancy remained. First, the sex of the journal's editor at the time of publication was strongly associated with the rate of women published. Second, there is a solid glass ceiling preventing women from publishing at similar rates to their male counterparts (McSweeney et al., 2000). A glass ceiling describes the experience of a woman in a male-dominated field being prevented from promotion, authority, raises, etc. compared to her male counterparts. The current study replicated and expanded McSweeney et al.'s work and aimed to find if publication rates have continued improving since the last study and if the sex of editors-in-chief mediated any discrepancy in publication rates by sex.

The disparity in published research between men and women spans many disciplines. Bendels et al. (2018) found that women were included in authorship in only 29.8% of nearly 300,000 articles across life sciences journals, multidisciplinary journals, Earth and environmental journals, and chemistry journals (totaling 54 journals between 2008 and 2016, many of which are male-dominated fields). In a similar study focusing on publication rates within primary health care and internal medicine journals, Sebo et al. (2020) found that women made up an average of 48% of authorship, but only 33% of articles specifically in the discipline of internal medicine. Lastly, in their dissertation examining female representation in disciplines related to working with individuals with intellectual disability^1^, Porter et al. (2003) found that females were included in 45% (with a range from 37 to 62%) of authorship. However, what sets this last finding apart from the first two is that the disciplines they focused on would all be considered female-dominated fields^2^ (i.e., social work, special education teaching, ABA).

Women are involved in academia nearly as much as men; however, women are represented less in higher levels (i.e., full professors) (Jones and Palmer, 2011) and in specific departments (National Center for Educational Statistics, 2010). Jones and Palmer (2011) summarized data from the National Center for Education Statistics (NCES) stating that women held 45.8% of academic positions compared to men, these figures represent an average of all departments. When broken down further, data show far more glaring disparities in female representation in science, technology, engineering, and math (STEM) fields, but only one department would be considered female-dominated (Table 1). Additionally, further examination of the data set showed a striking difference between the rank of males and females in postsecondary education. Male full professors in the United States made up 30% of the total male faculty, yet female full professors made up only 15% of the total female faculty (National Center for Educational Statistics, 2010, p. 365).

**Table 1:** Percentage of gender differences in professorship by department, Fall 2003.

**Department**	**Male**	**Female**
Natural sciences	74.5	25.5
Social sciences	64.3	35.7
Engineering	91.5	8.5
Business	68.5	31.5
Education^a^	39.3	60.7
All departments	52.1	47.9

*This table was adapted from summarized data provided by National Center for Educational Statistics (2010)*.

a *Education is the only department in which female faculty represented at least 60% of the total faculty*.

Based on the data found by National Center for Educational Statistics (2010) women are much less likely to be tenured or in tenure-track positions than men, but even so, the process of *promotion* (i.e., finding out about potential promotion opportunities, or meeting the expectations for consideration of promotion) to full professor is more difficult for women across universities (Dolan, 2009). There are likely many reasons that this disparity exists and one aspect that has been studied has to do with an aspect of the “tenure trifecta” (Deo, 2018, p. 1022). The tenure trifecta consists of teaching, service, and scholarship (Deo, 2018, p. 1022). In their review of literature studying tenure processes across universities, Schimanski and Alperin (2018) found that while all three aspects of the trifecta are considered in the tenure process, research/scholarship has recently become a heavier weight during consideration.

Previous studies have reviewed various statistics regarding female editors. Some research all members of an editorial board, whilst others focus on editors-in-chief or co-editorships. These definitions make the current study's findings difficult to compare to previous studies. In the original study, McSweeney et al. (2000) focused on the makeup of editorial boards with females representing between as few as 19% in 1978 to as high as 29% in 1997. Porter et al. (2003) found 3–51% of board members were female. McSweeney and Parks (2002) saw 11% females on editorial boards in 1978 increased to 46% in 1998. Editorial boards were not reviewed in this study, but since it is difficult to find studies that focus on female editors-in-chief, it's important to look at these broader data points. This is an area that would benefit from future study.

While sex distinctions in academic publications in general is an important aspect of research, this research focuses on one female-dominated field of science called ABA. This field is specifically focused upon due to (1) the nature of the researcher's background in the field, and (2) the fact that the field is dominated by women. Further, the researcher found it important to continue research where McSweeney et al. (2000) left off. Based on these issues, the researcher sought to answer the following questions:

1. Are women represented, overall, more frequently than in previous studies?
2. Are women represented in the first author position at a lower rate than men?
3. Are women represented in the last author position at a lower rate than men?
4. Are women published more frequently when the editor of the journal is a woman?

## Methods

The researcher utilized a data scraping operation of all articles published in eight prominent behavior analytic journals. One additional journal, *Journal of Applied Behavior Analysis*, was subsequently excluded due to problems related to the data scraping tool being unable to reliably recognize the code used in that journal. The researcher examined the remaining seven journals for similar issues and was unable to find any problems with the instrument.

### Publications

To best identify any significant differences in publication based on sex of the article authors, the researcher reviewed seven journals. They were chosen for four reasons: (1) they are the more prominent of journals in the field of ABA based on their association with the field's most prolific professional organization (Association for Behavior Analysis International); (2) they are easily accessible—available online; (3) they have existed *throughout* the intended review span; and (4) this list contains all of the journals reviewed in the original research that has been replicated and expanded upon (except for *Journal of Applied Behavior Analysis*). These journals were scoured for all articles published between 1997 (when the previous study ended) and 2019 (the year prior to this research beginning). Although not perfectly divisible by five, the author presents the data in 5-year intervals (except the most recent interval includes only 3 years) to synthesize the data more effectively to show changes and to mirror the procedure used in the previous study.

### Procedures

The researcher ran a Boolean phrase search within the Ebscohost database to find all published articles within a given journal, including all volumes and issues between January 1997 and December 2019. Once all articles were identified, the citations were exported into a CSV file, transferred and organized in an Access file, scraped for necessary information, exported to an Excel file, and finally coded. The information coded included: name of journal, year of the journal, name of the article, sex of each author, and order of each author listed (and this information was repeated for each of the seven journals, for all included years/volumes/issues). The authors (*N* = 27,225) were separated and further coded.

The names of the article authors were categorized into “first authorship” “last authorship” and “included in authorship.” Once categorized by order of authorship, the authors were input into a gender predictor tool called genderizeR (Wais, 2016). This particular package has been shown to have the most accurate classification rate and “smallest gender bias” compared to other gender prediction services (Santamaría and Mihaljević, 2018, p. 24). Each author was assigned the label of either “female” or “male” along with a probability rating between 0 and 1 (0 being 0% accurate and 1 being 100% accurate). The tool was able to code the majority of author names (*n* = 23,958) with at least 80% accuracy. The researcher manually coded any author name that fell between 0 and 0.79 accurate (*n* = 3,267). If the author's name was readily associated with a particular sex, the researcher coded it and moved on. Examples of names that are readily associated with sex are Sarah (female), Robert (male), Jennifer (female), or Jacob (male). If the author's name was not readily associated with a particular sex, further research was used to determine the author's sex (*via* Google/Google Scholar search, University website search, searching Scopus, and/or looking through the ORCID registry). Examples of names that were not readily associated with sex, requiring further investigation, include Dani, Kelly, Zao. The researcher examined references to the individual's pronouns, if found, or pictures of the individuals to sort them into “female,” “male,” or “unknown.” Of the 12% of authors with <0.80 probability, the researcher was able to code 42% into “female” or “male” (*n* = 1,372), labeling the remaining 58% (*n* = 1,895) as “unknown.”

Editor names were collected from a combination of procedures. First, as many editors as possible were coded after searching through the publisher websites. If the information wasn't readily available, the following steps were taken (in order) to determine the editors from 1997 to 2019: (a) searching through the individual journal's website, (b) searching across professional associations, (c) reviewing editorials in various volumes and issues of the journal, (d) emailing current editors for information on past editorships, and (e) emailing a collection of editorial board members to help fill in any leftover gaps. *Behavior Modification* was the only journal to fail to yield editor names (this journal was utilized in the data analysis for female participation and order of authorship, but editor/author relationships were not possible to analyze). The sex of the editor of the journal at the time of publication was determined using the same methods described previously for manually coding the sex of the article authors.

## Results and Discussion

Current practitioners in the field of ABA are made up of 88% females (Nosik et al., 2018). Previous research in the field of ABA showed that female academic authors were published a fraction as often as male authors (McSweeney et al., 2000). This discrepancy has been shown repeatedly in the literature, the makeup of ABA companies, and in higher education teaching (McSweeney and Swindell, 2001; Myung et al., 2011; Pyke, 2014; Winchester and Browning, 2015; Helmer et al., 2017). The current study was created to determine if the discrepancies in article publications have continued in recent years compared to the original McSweeney et al. (2000) study.

The researcher examined the publication rates of female journal authors across seven journals between 1997 and 2019 (Table 2). Once all data was downloaded, coded, and sexed, the researcher created graphs to visually show the differences in publication rates by journal and over time. Time was divided into 5-year intervals through 2016. 2017–2019 was included as a “5-year interval” since data collection consisted of all articles up to the end of 2019. Certain relationships were tested in SPSS and are included with the visualization graphs. The intervals created were arbitrary and the statistics do not appear to have been effected by having one shorter interval. Variables compared include the name of the journal, year of the article, authors' sex, editors' sex at the time of publication, and authorship position.

**Table 2:** Journals, articles, and authors studied.

**Journal name**	**Journal abbreviation**	**Impact factor (most recent)**	**Total articles**	**Total authors**
The analysis of verbal behavior	AVB	unpublished	216	905
Behavior modification	BM	2.024	1,270	3,571
Behavior therapy	BT	3.228	1,693	5,585
Behavior research & therapy	BRT	4.134	3,128	11,427
Education & treatment of children	ETC	1.24	708	2,164
Perspectives on behavior science; the behavior analyst^a^	PoBS/BA	1.357	518	510
The psychological record	TPR	1.026	1,252	3,063
	Total		8,785	27,225

a *The Behavior Analyst was renamed to Perspectives on Behavior Science in 2018*.

### Overall Female Representation

**Figure 1:**
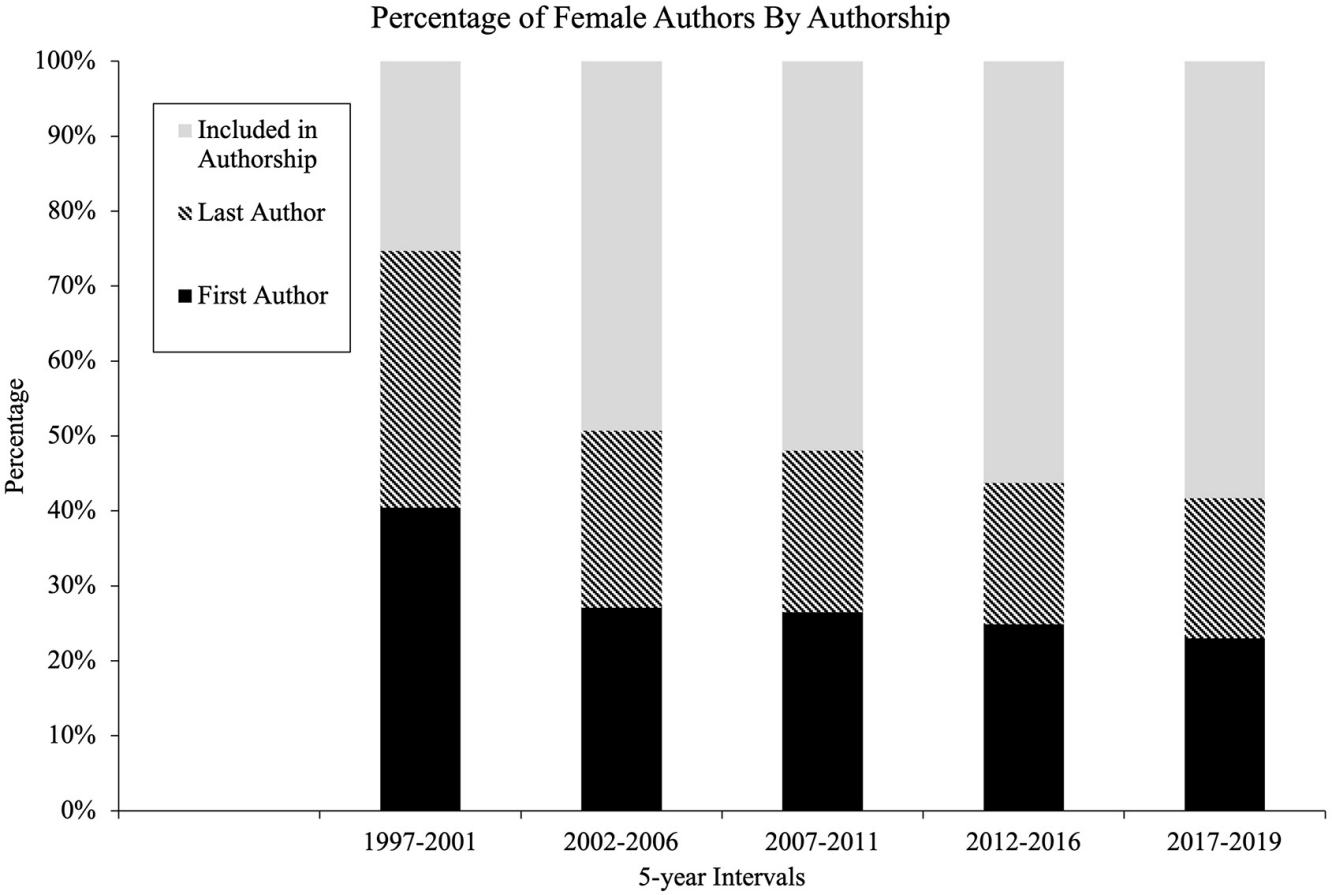
Total percentage of female authors over time by journal.

The number of female authors published between 1997 and 2019 increased in all journals. Some journals showed a more drastic improvement in the rate of publication by females from 37 to 63% of authors and 28–56% (BM and AVB, respectively), others improved only slightly from 30 to 39% and 25 to 35% (TPR and PoBS/BA, respectively). When comparing rates of female authors with the original study (McSweeney et al., 2000), larger changes are visible. BM, BT, and BRT increase female authorship from 24, 23, and 22% (respectively) to 63, 55, and 51% (respectively) (Figure 1).

The visual changes over time sparked further statistical investigation. Chi-square and Cramer's V statistics were run to calculate the significance between sex and interval of time. Chi-square statistics revealed a significant relationship (*p* < 0.001) between sex and all intervals (Table 3). The Cramer's V statistic was used to determine the level of strength of the relationship. Based on the degrees of freedom associated with the statistics, a Cramer's V statistic of 0.06–0.16 is a small effect size, 0.17–0.28 is a medium effect size, and 0.29 and higher is a large effect size (Pallant, 2016). A small effect size was observed for all intervals: 1997–2001 (0.108), 2002–2006 (0.150), 2007–2011 (0.122), 2012–2016 (0.112), 2017–2019 (0.139), and in total (0.116).

**Table 3:** Chi-square and Cramer's V statistics comparing author gender by year group.

**Publication year**	**N of valid cases**	**Chi-square**			**Cramer's V**
		**Value**	**Significance**	**df**	
1997–2001	3,000	35.042	0.000	6	0.108^*^
2002–2006	4,231	94.920	0.000	6	0.150^*^
2007–2011	5,875	86.903	0.000	6	0.122^*^
2012–2016	6,984	88.043	0.000	6	0.112^*^
2017–2019	4,891	94.552	0.000	6	0.139^*^
Total	24,981	335.621	0.000	6	0.116^*^

* *Small effect size (Pallant, 2016)*.

Seven journals were reviewed for female authorship. Across these seven journals, each one showed an increase in the percentage of female authors vs. male authors across every time interval. Initial analysis seemed relatively positive, but further investigation yielded more concerning figures. First, while increases have occurred, there were instances in which the percentage of female authors had increased higher in middle year groups and then decreased again by 2019 (Figure 1). *Behavior Therapy* (BT) increased to 54% in 2007–2011, dropped to 50% in 2012–2016, before increasing to 55% in the most recent interval. *Behavior Research and Therapy* (BRT) increased from 36% in 1997–2001 to 52% in 2012–2016, before falling back to 51% in 2017–2019. Similar results were found for *Education and Treatment of Children* (ETC), *The Analysis of Verbal Behavior* (AVB), and *The Psychological Record* (TPR). The trend of overall female representation was consistently upward across all journals and 5-year intervals. For some of the journals, *Behavior Modification* (BM), BT, ETC, and AVB, the percentage of female authors is relatively close to the percentage of board-certified females in ABA, especially those designated at the doctoral level (68.3%) (Li et al., 2018). However, *Perspectives on Behavior Science/The Behavior Analyst* (PoBS/BA) and TPR have much greater steps to take to come close to that number. PoBS/BA is comprised of only 35% and TPR only 39% female authors in the most recent year group (2017–2019).

### Females Across Authorship Positions

**Figure 2:**
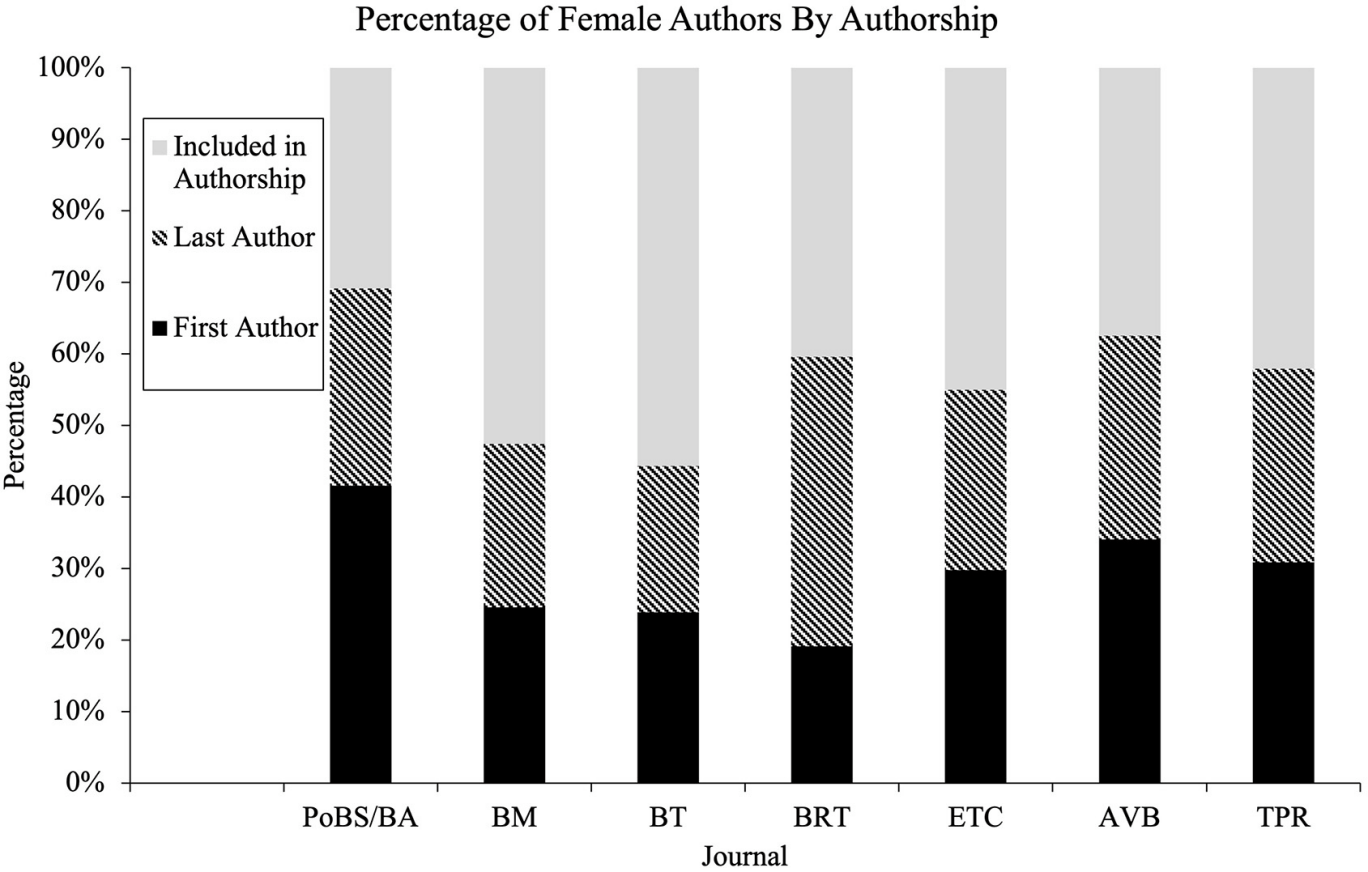
Percentage of female authors by authorship positions by year.

While the data shows that female publication has increased steadily over the last few decades, females in ABA have been featured as the first and last author at a steadily *declining* rate (Figure 2). From 1997 to 2001, female authors were in the first author position in 40% of articles, last author position 34% of articles, and included in authorship 25% of articles. However, by 2017–2019 female authors were represented in the first and last author positions 23 and 19% of the time.

**Figure 3:**
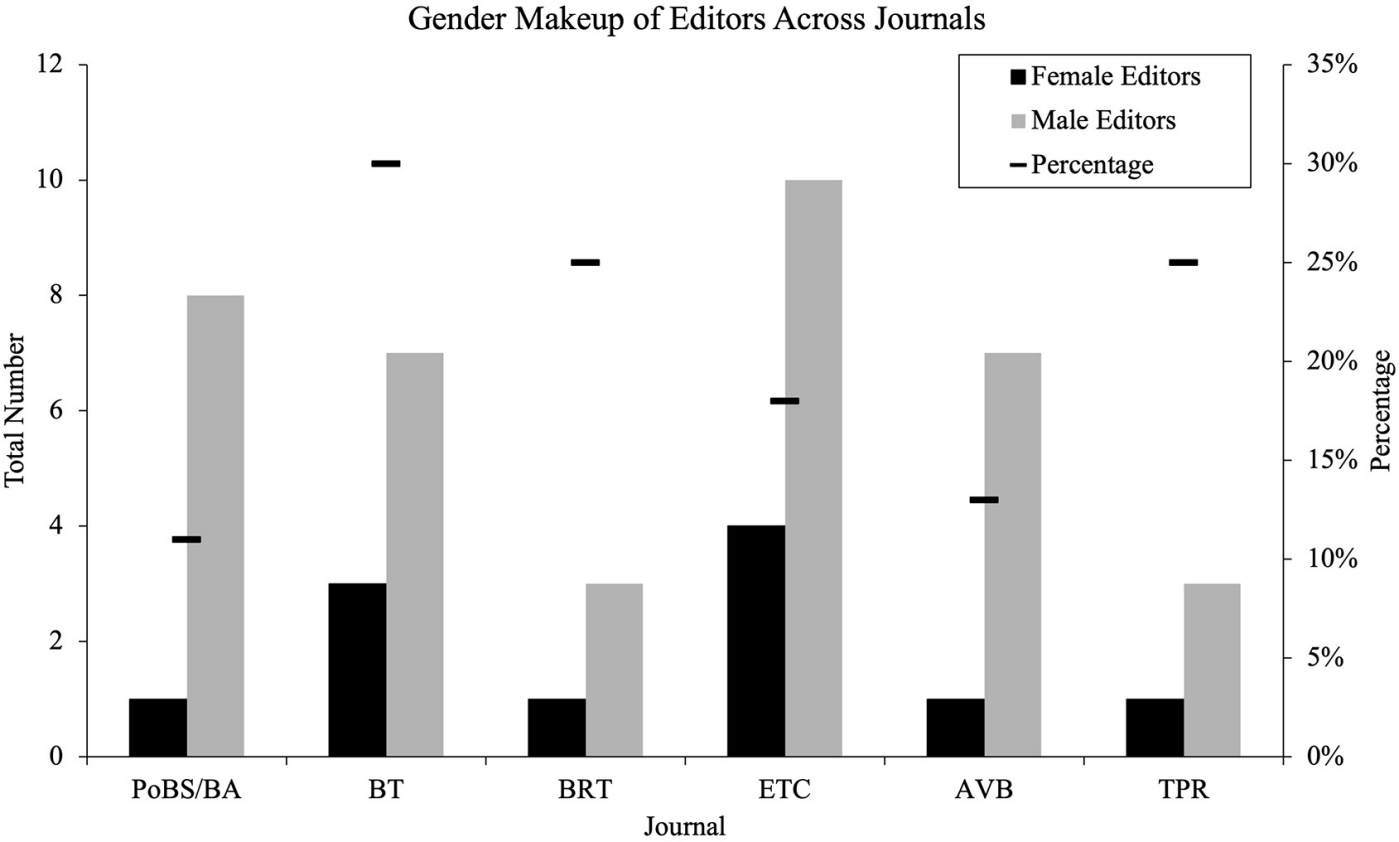
Percentage of female authors by authorship positions by journal.

Breaking the order of authorship down by journal yielded interesting results. BRT was the journal with the highest percentage of female authors; however, they had the lowest percentage of female first authors. PoBS/BA has the smallest percentage of female authors; however, they had the highest percentage of female first authors (Figure 3).

Discrepancies in male/female authorship is only part of the problem. The order of authorship is of importance to researchers inside and outside of academia for name recognition, prestige, for upward mobility in their career (McSweeney and Parks, 2002; Porter et al., 2003; de Camargo and Hayashi, 2017). It is more prestigious for an author to be in the first position than in the middle—and in the field of ABA, the last position is now reserved for the more prestigious member of the team (advisor, mentor) rather than solely by order of contribution (Li et al., 2018). This study shows a drastic change in the proportion of first, last, and included authors. Included female authors have increased steadily over time. However, the number of first and last authors have drastically decreased.

When layered by year, Chi-square revealed a significant relationship (*p* < 0.001) between sex and order of authorship in every 5-year interval (Table 4). A small effect size was observed for all intervals except the most recent interval: 1997–2001 (0.069), 2002–2006 (0.077), 2007–2011 (0.063), 2012–2016 (0.072), and in total (0.071). When layered by journal, Chi-square revealed a significant relationship between sex and order of authorship in every journal (AVB- *p* = 0.008; ETC- *p* = 0.001; *Behavior Modification* (BM), *Behavior Therapy* (BT*), Behavior Research and Therapy* (BRT), *Perspectives on Behavior Science/Behavior Analyst* (PoBS/BA), *The Psychological Record* (TPR), and in Total- *p*< *0*.001) (Table 4). The Cramer's V statistic was used to determine the level of strength of the relationship. Based on given degrees of freedom, a small effect size was observed for AVB (0.116), BRT (0.075), ETC (0.065), PoBS/BA (0.143), TPR (0.085), and in total (0.071). A statistically significant effect size was not observed for BM and BT.

**Table 4:** Chi-square and Cramer's V statistics comparing author gender and authorship position by year group and journal.

**Publication year and journal name**	***N* of valid cases**	**Chi-square**			**Cramer's V**
		**Value**	**Significance**	**df**	
1997–2001	3,118	29.285	0.000	4	0.069^*^
2002–2006	4,643	55.452	0.000	4	0.077^*^
2007–2011	7,027	56.141	0.000	4	0.063^*^
2012–2016	7,450	76.904	0.000	4	0.072^*^
2017–2019	4,987	24.767	0.000	4	0.050
AVB	509	13.673	0.008	4	0.116^*^
BM	3,771	23.895	0.000	4	0.058
BT	5,585	38.624	0.000	4	0.059
BRT	11,426	128.632	0.000	4	0.075^*^
ETC	2,166	18.074	0.001	4	0.065^*^
PoBS/BA	905	31.199	0.000	4	0.143^*^
TPR	3,063	44.583	0.000	4	0.085^*^
Total (years)	27,225	273.372	0.000	4	0.071^*^
Total (journals)	27,225	273.372	0.000	4	0.071^*^

* *Small effect size (Pallant, 2016)*.

In 1997–2001, female authors were represented in the first author position in 40% of the articles including female authors. By 2017–2019 this number dropped to only 23% of the articles including female authors. Last authorship reduced from 34% of articles to 19%. But overall, that percentage went from 26% of all articles to 34%. These relationships were statistically significant for all year groups (*p* < 0.001) and small effect sizes were observed for intervals; similar findings were found for each individual journal (*p* < 0.001) with a small effect size observed for five of seven journals. Previous studies found that female first authors increased steadily over time, 10–65% (McSweeney and Parks, 2002) or across journal from 25 to 46% (Li et al., 2018), 27–57% (de Camargo and Hayashi, 2017), or 33–55% (Porter et al., 2003). While average participation increased for female authors, the more prestigious authorship positions were much more likely to be occupied by male authors.

### Sex of the Editor

This study focused on the percentage of articles that were published under each editor, and their corresponding data related to author sex, but also included a review of the total number of editors in chief over time and journal. In the journals with the highest proportion of female editors, representation was only 24.6–39.4%, depending on the journal. Helmer et al. (2017) found a range between 6 and 37% depending on the journal. The current study found similar proportions of female editors-in-chief (or co-editors) to previous studies, but still aren't close to parity to that of male editors. PoBS/BA and AVB showed the smallest proportions of female editors at 11 and 13% respectively. TPR and BRT had 25% of their editorships held by females, falling well below BT at 30% female editors-in-chief. In all, a mean of 20% of editorships were held by female editors.

**Figure 4:**
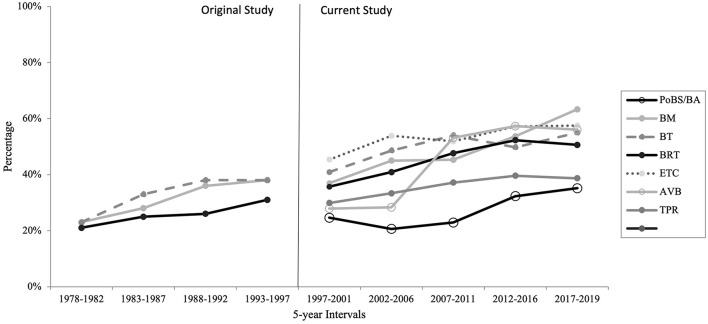
Gender makeup of editors by journal.

The number of female editors (head editor/editor-in-chief/co-head editor) shows a disparity between female and male editors. Depending on the journal, female editors are represented up to 30% of editors (as in BT) or as low as 11% (as in PoBS/BA). Within the time frames and across the seven journals, female editors make up an average of 20% of all editorships (Figure 4). Most journals have only had one female editor in the 23-year span. While ETC showed the largest proportion of female editors, the co-editorships made the data more difficult to analyze, so the percentage of female editors for each year were averaged together to obtain a more accurate percentage of female editors (18%).

### Sex of the Editor at the Time of Publication

The sex of an editor at the time of publication seems to play a significant role in the rate of publication by female authors. The percentage of female editors was relatively low from 1997 to 2011. This coincides with the relatively lower percentage of female authors published in the same time period wherein five of the seven journals surpassed the 50% mark for female authors. From 2012 to 2016, female editorship increased from 8 to 43% and increased further to 71% in 2017–2019.

When layered by year, Chi-square revealed a significant relationship between author sex and editor sex in 1997–2001 (*p* = 0.006) and all other 5-year intervals (*p*< *0*.001) (Table 5). A small effect size was observed for all intervals except the oldest interval: 2002–2006 (0.097), 2007–2011 (0.141), 2012–2016 (0.134), 2017–2019 (0.078), and in total (0.113).

**Table 5:** Chi-square and Cramer's V statistics comparing author gender and editor gender by year group and journal.

**Publication year and journal name^a^**	**N of valid cases**	**Chi-square**			**Cramer's V**
		**Value**	**Significance**	**df**	
1997–2001	3,118	14.484	0.006	4	0.048
2002–2006	4,643	87.706	0.000	6	0.097^*^
2007–2011	7,027	280.214	0.000	6	0.141^*^
2012–2016	7,450	265.882	0.000	6	0.134^*^
2017–2019	4,987	61.171	0.000	6	0.078^*^
Total (year)	27,225	696.473	0.000	6	0.113^*^
AVB	509	2.259	0.323	2	n/a
BT	5,585	773.136	0.000	2	0.372^**^
BRT	11,426	132.345	0.000	2	0.108^*^
ETC	2,166	5.747	0.056	2	n/a
PoBS/BA	905	4.455	0.108	2	n/a
TPR	3,063	19.517	0.000	2	0.079^*^

a *Behavior Modification was excluded due to a lack of editorial data*.

* *Small effect size (Pallant, 2016)*.

** *Large effect size (Pallant, 2016)*.

Interesting results were found when isolating the journal to find patterns in publication depending on the number of female editors. *Perspectives on Behavior Science/Behavior Analyst* (PoBS/BA) had the lowest percentage of female editors and the lowest percentage of female authors. However, *Analysis of Verbal Behavior* (AVB) had the second lowest percentage of female editors, yet the highest increase in female authorship over time, while *The Psychological Record* (TPR) has the highest percentage of female editors and the lowest increase in female authorship over time. Of all journals, TPR has had the highest percentage of female-only editors and *Education and Treatment of Children* (ETC) has had the highest percentage of female editors in total (due to frequent multi-sex co-editorships).

When layered by journal, Chi-square revealed a significant relationship between author sex and editor sex in half of the journals that reported the sex of the editors (BT, BRT, TPR all at *p*< *0*.001) (Table 5). AVB, ETC, and PoBS/BA did not show statistically significant relationships between author and editor sex. The Cramer's V statistic was used to determine the level of strength of the relationships for BT, BRT, and TPR. Based on given degrees of freedom, a small effect size was observed for BRT (0.108) and TPR (0.079); and a large effect size (0.372) was observed for BT.

This lack of female representation has an impact on the publication of female authors. In the original study, McSweeney et al. found that “women were substantially more likely to participate as authors when articles were edited by a female editor” (2000, p. 274). Data analysis showed that the sex of the editor was significantly related to the sex of the author in half of the journals with editorship data: *Behavior Therapy* (BT), *Behavior Research and Therapy* (BRT), and *The Psychological Record* (TPR) (*p*< 0.001); *Perspectives on Behavior Science/Behavior Analyst* (PoBS/BA) approached significance (*p* = 0.056). A small effect size was observed for BRT and TPR and a large effect size was observed for BT. BRT showed a large percentage of female editors and female authors showing that the relationship between editor and author sex resembles that of the original study. TPR had a fewer percentage of female authors and a moderate percentage of female editors, which varies a bit from the original study. However, BRT had both the highest percentage of female authors [behind *Behavior Modification* (BM), which was excluded from this particular analysis due to having no editorial data] and the highest percentage of female editors. These findings mirror the original study, at least for half of the journals reviewed.

Examination of the percentage of female authors and female editors over time yielded similar results to the original study. 1997–2001 shows the fewest number of female authors and a low percentage of female editors, while 2012–2016 and 2017–2019 show the highest percentages of female authors and editors. This relationship still exists between 2002–2006 and 2007–2011, but at this point the percentage of female editors shifts to a lower percentage of female editors but a larger percentage of female and male co-editorships. Each year group yields a statistically significant relationship (*p* < 0.001) and all but one (1997–2001) is supported with a statistically significant effect size. The original study did not include distinctions or relationships over time, only by a single journal. More research is needed to better understand this complicated relationship and whether having blind or double-blind peer-review could mediate some of these problems in publication (Helmer et al., 2017). It is clear that there is a relationship between the sex of the author submitting an article and the sex of the editor at the time of publication.

## Conclusion

Overall, this research reflected similar results to the previous study along with interesting findings differentiating this research from previous literature. Consistent with vast research on the authorship of females vs. males (Bardolph and Vanderwarker, 2016; de Camargo and Hayashi, 2017; Tushingham et al., 2017; Bendels et al., 2018; Li et al., 2018), this research found that males are published, in first author positions, and are editors in chief much more frequently than females. Visual data showed that there has been upward growth in the rate of publication of female authors within the female-dominated field of ABA. Statistics confirmed the relationship between author sex and (a) time, (b) journal, and (c) order of authorship. Some relationships were found between author sex and editor sex. Order of authorship showed significance across all year groups and journals and, in all but two relationships, a small effect size was observed. Overall female authorship has increased since 1997, yet the more prestigious authorship positions (first and last) have decreased over time. While some journals have increased their number of female authors more than 25% (BM and AVB), others have much more room to grow in becoming more equitable in terms of sex (TPR and PoBS/BA). Further, a journals' impact score does not appear to be related to the publication outcomes or in changes of participation over time.

The number of board-certified females in applied behavior analysis (ABA) is not equitable to the number of female authors compared to male authors across various journals and over time (although the number of female authors has drastically increased over the years) (Nosik and Grow, 2015; Nosik et al., 2018). That said, “women are substantially underrepresented as both authors and editors” (Li et al., 2018, p. 163) and continue to be underrepresented as shown by the results of the current study. Both female authorship and female editorship was found to be inequitable compared to male authorship and editorship. Further, female authors in more prestigious authorship positions (i.e., first and last) are much less represented than male authors in those authorship positions.

It is nearly impossible to determine the reason for such discrepancies, both between each of the journals and from participation in the field compared to participation in academic publication. Female authorship at a proportionally lower rate than their participation in other aspects of their respective fields could be due to a higher likelihood to pursue applied careers instead of academic ones that would necessitate a higher push toward publication (Kessler et al., 2014), a career that requires publication may not be as attainable for those that are interested in starting or caring for a family (de Camargo and Hayashi, 2017), intentional or unintended occupational segregation by sex, putting females at a disadvantage toward pursuing publication (Burkinshaw and White, 2017), or female professionals choosing to work outside of research-focused universities, where publication is less emphasized (i.e., state colleges) (Parker, 2015). Whatever the cause, it is something to be investigated further to find more specific areas to improve.

The current study showed positive increasing trends for female authors publishing articles in academic journals in ABA. First, inclusion in authorship has increased between 1997 and 2019 and in each of the seven journals reviewed. These results mirrored the findings of McSweeney et al. (2000). Second, female editorship has increased between 1997 and 2019. This is an aspect that was not included in the original study (as they focused on the sex makeup of the editorial board, rather than the editor-in-chief).

The current study also found several concerning figures in the participation by women in behavior analytic journals. First, while authorship in general has increased, the more prestigious first and last author positions have seen a drastic decrease in female authors. These findings were similar to those found by McSweeney et al. (2000). Second, some of the journals included very few female editors (as low as 11% of the articles compared to those edited by male editors). This finding isn't surprising considering that the original study showed that “women were more likely to appear as authors than as first authors and as first authors than as members of the editorial board (a glass ceiling)” (p. 275).

Lastly, this study expanded on the original study to find more data pertaining to the relationship between author sex and editor sex. The original study found that female authors were more likely to be published if the editor was also female (and female editors more likely to publish articles with female first authors). While this study did not look at the relationship between order of authorship and editor sex, there was a significant relationship between female authors and female editors over time and across half of the journals. The glass ceiling has yet again shown to exist and impact the academic literature available to those in ABA, just as the glass ceiling has impacted females across various fields (Organisation for Economic Co-Operation Development, 2020).

## Limitations and Recommendations for Further Study

The genderizeR (Wais, 2016) is an AI software that predicts the sex of an individual *via* likelihood. There is a chance that the names coded with 80% confidence or higher were incorrectly coded. However, Wais (2016) found that the software was slightly more accurate than two comparison studies in which sex was hand-coded by the authors. Further, as discussed earlier, there is a possibility that the authors would not have associated themselves with the category that they were coded into, even if their name were readily associated with a specific sex. Manual coding was difficult for those authors that used only initials (especially when paired with a common surname). If the researcher couldn't find definite proof of the author's sex, they were coded as “unknown.” To help the researcher find the most likely category of sex when manually coding, they looked for instances in which the authors were mentioned with specific pronouns and used that information to help code the ambiguous or androgynous names. In addition, sticking to a binary coding format was done strictly to mimic the previous study, but may not be inclusive of individuals that prefer another gender categorization (non-binary, intersex, agender, gender neutral, or gender fluid).

There is a possibility that the names that were manually coded naturally led to more female authors being coded. Since hyphenated last names are more likely to belong to women (Shafer and Christensen, 2018) they are more likely to be unique, giving the researcher a higher likelihood of being able to find them through various manual searches to confirm their sex.

This study focused on a small subfield of psychology.

ABA is a growing field, and a follow up study was necessary to track publication rates over time since McSweeney et al. (2000) published their original study. However, ABA is a very small niche field within the realm of human behavior. It is possible that the findings for this field won't be seen in the broader fields, making it less generalizable. A larger scale study across disciplines may give a more accurate representation of women's participation in publications more broadly, especially in those that would be considered female-dominated (i.e., teaching, nursing, psychology).

In addition to sex equity, racial and ethnic equity is something our society values. While the data may be more difficult to obtain, systematic analysis of journals' inclusion of diverse authors is necessary across scientific fields. It is crucial for future research to study the publication rates of people of color (POC) in ABA and beyond. If the potential problem isn't first identified, the potential solutions cannot be created and implemented to address such problems.

This research included seven widely known journals. However, there was one journal that was removed from the study. When articles from the *Journal of Applied Behavior Analysis* (JABA) were downloaded, it was noted that several volumes were missing while several articles were duplicated but showed inconsistent DOI numbers. In order to maintain validity to the study, JABA was not included in this study. Future studies should include JABA in the comparisons.

Another area that was not included in this study was the sex makeup of editorial boards. This information may yield important information on the participation of female authors. Further, more information regarding the sex of editors-in-chief compared to the sex of editorial board members is needed to give researchers a full picture of the relationship of sex in academic publication processes. Although this study was intent on focusing on the first wave of subjective determination of a study's appropriateness in a journal, meaning the focus needed to be on the sex of the first reviewer (which is most often the journal's editor-in-chief).

This research focused on the variables first studied in McSweeney et al. (2000) but some key variables would help to better contextualize the results. Further research should include information related to whether or not article submissions are reviewed blind (or double blind) and where in the submission process is the author's identifying information made available. These variables could better explain the variations in female authorship across journals.

## Data Availability Statement

The datasets presented in this study can be found in online repositories. The names of the repository/repositories and accession number(s) can be found below: Sac State Scholars, https://hdl.handle.net/20.500.12741/rep:9.

## Author Contributions

The author confirms being the sole contributor of this work and has approved it for publication.

## Funding

This work has been recycled from an approved and complete thesis at California State University, Sacramento.

## Conflict of Interest

The author declares that the research was conducted in the absence of any commercial or financial relationships that could be construed as a potential conflict of interest.

## Publisher's Note

All claims expressed in this article are solely those of the authors and do not necessarily represent those of their affiliated organizations, or those of the publisher, the editors and the reviewers. Any product that may be evaluated in this article, or claim that may be made by its manufacturer, is not guaranteed or endorsed by the publisher.

## Footnotes

^1^Since the publication of the article referenced, the term “mental retardation” has been replaced with the term “intellectual disability”.

^2^For consistency, the author refers to any field as “female-dominated” if women make up at least 60% of the workforce.
